# Parameterization of the Durations of Phases of Foot-And-Mouth Disease in Cattle

**DOI:** 10.3389/fvets.2019.00263

**Published:** 2019-08-09

**Authors:** Shankar Yadav, Carolina Stenfeldt, Matthew A. Branan, Karla I. Moreno-Torres, Lindsey K. Holmstrom, Amy H. Delgado, Jonathan Arzt

**Affiliations:** ^1^Foreign Animal Disease Research Unit, Plum Island Animal Disease Center, Agricultural Research Service, United States Department of Agriculture, Greenport, NY, United States; ^2^Monitoring and Modeling, Center for Epidemiology and Animal Health, Animal and Plant Health Inspection Service, United States Department of Agriculture, Fort Collins, CO, United States; ^3^Plum Island Animal Disease Center Research Participation Program, Oak Ridge Institute for Science and Education, Oak Ridge, TN, United States; ^4^Department of Veterinary Population Biology, University of Minnesota, St. Paul, MN, United States

**Keywords:** foot-and-mouth disease, FMD, virus, FMDV, epidemiology, parameterization, modeling, cattle

## Abstract

The objective of the current study was to update parameterization of mathematical simulation models for foot-and-mouth disease (FMD) spread in cattle utilizing recent knowledge of FMD virus (FMDV) pathogenesis and infection dynamics to estimate the duration of distinct phases of FMD. Specifically, the durations of incubation, latent, and infectious periods were estimated for 3 serotypes (O, Asia1, and A) of FMDV, individually and collectively (pan-serotypic). Animal-level data were used in Accelerated Failure Time (AFT) models to estimate the duration of the defined phases of infection, while also investigating the influence of factors related to the experimental design (exposure methods) and virus serotype on disease progression. Substantial influences upon the estimated duration of distinct phases of FMD included the quantity of viral shedding used as a proxy for the onset of infectiousness, virus serotypes, and experimental exposure methods. The use of detection of any viral RNA in nasal secretions as a proxy of infectiousness lengthened the total infectious period compared to use of threshold-based detection. Additionally, the experimental system used to infect the animals also had significant effects on the duration of distinct phases of disease. Overall, the mean [95% Confidence Interval (CI)] durations of pan-serotype disease phases in cattle were estimated to be: incubation phase = 3.6 days (2.7–4.8), latent phase = 1.5 days (1.1–2.1), subclinical infectious phase = 2.2 days (1.5–3.5), clinical infectious phase = 8.5 days (6.2–11.6), and total infectious phase = 10.8 days (8.2–14.2). This study highlights the importance of identifying appropriate proxy measures to define the onset and duration of infectiousness in FMDV-infected cattle in the absence of actual transmission data. Additionally, it is demonstrated herein that factors associated with experimental design, such as virus exposure methods, may significantly affect disease progression in individual animals and should be considered when data is extrapolated from experimental studies. Given limitations in experimental data availability, pan-serotypic parameters which include all routes of exposure and a threshold-defined onset of infectiousness may be the most robust parameters for exploratory disease spread modeling approaches, when information on the specific virus of interest is not available.

## Introduction

Foot-and-mouth disease (FMD) is caused by FMD virus (FMDV) of family *Picornaviridae* (Genus: *Aphthovirus*) and is a contagious disease of cloven-hoofed domestic and wild animals (1–3). FMDV exists as seven serotypes (O, A, C, Asia1, SAT1, SAT2, and SAT3), with several strains and lineages within each serotype that vary in antigenicity, host-range, and disease dynamics (4). Currently, several countries in Asia, Africa, and Latin America are endemic for FMD (5, 6). In the United States, the most recent outbreak of FMD occurred in 1929 (7). However, FMD is still considered to be a high risk disease due to the massive implications associated with a potential incursion. Additionally, the ever-increasing globalization of human movement and commerce and the potential threat of bioterrorism accentuate the importance of maintaining preparedness for FMD outbreak response in countries that are currently free of the disease.

An outbreak of FMD would have severe adverse impacts on the economy of the U.S. livestock industry due to direct losses and the imposed domestic and international restrictions on trade and movements of livestock and related commodities (4, 8, 9). Furthermore, an FMD epidemic would require significant resources for control and eradication. Planning for the control of an FMD outbreak in the U.S. must take into account the underlying complexities of FMDV dynamics within specific host species and the rates of dispersion within the naïve U.S. livestock and wildlife populations. These factors are likely to affect the effectiveness of complex and coordinated disease control strategies which may include mass depopulation (stamping out), movement restrictions, controlled marketing, and vaccination (3, 10–12). Understanding how a pathogen may spread and evaluating the effectiveness of possible control measures can strengthen preparedness plans and help to inform resource decisions, reducing strategic, and logistical complications in early response (13, 14).

Mathematical modeling tools are often employed to assess the impacts of a potential FMD outbreak in a disease-free country (15, 16). However, FMD simulations with biologically implausible disease-related parameterizations may result in flawed predictions and misguided control efforts, which has been suggested in association with the 2001 FMD outbreak in the United Kingdom (17). Unfortunately, determining the duration of the various sequential and distinct phases of infection can be challenging when experimental data is limited and inconsistent. Detailed epidemiologic data required to estimate these phases are rarely available from outbreaks, but transmission experiments carried out in high-containment research facilities can be a useful source of data for estimating disease parameters (18). Given the diversity in pathogenicity of FMDV strains, efforts to develop epidemiologic parameters need to account for both strain and host species-specific characteristics in order to reasonably estimate spread within a population (17, 19). Because of the great diversity of pathogenicity across FMDV strains, unified epidemiological parameter estimates at the pan-serotype level could be useful for parameterization of FMD disease spread models to support planning and preparedness. When an outbreak occurs, it is impossible to predict the behavior of a novel strain, even when the serotype is identified. In addition, when basing parameterization upon data from experimental studies, aspects of the experimental design such as the route and dose of virus exposure may also impact the disease dynamics within the animal. For example, for FMD in cattle, it was shown that direct injection-based inoculation evaded critical components of the host immune system, and thereby led to rapid disease progression within the animals (20). Contrastingly, virus exposure through direct or indirect contact with other infected animals may lead to slower disease progression, while the efficiency of transmission may also be affected by the animal species that is the source of the virus (20). Parameters which take into account this diversity in experimental methods and viral strains can serve as a useful starting place for estimating the impacts of FMDV introduction and the effectiveness of alternative control strategies.

Recently published studies focused on deriving epidemiologic parameters for FMD have had to rely on numerous assumptions due to limitations in data availability or structure (21). In particular, contagiousness has been defined based upon detection of viral RNA in either tissues or secretions by rRT-PCR, which has become the most commonly used manner of detecting FMDV. However, various studies have demonstrated that detection alone of FMDV RNA in secretions or tissues of cattle does not consistently correlate with presence of infectious virus (20, 22–24). Additionally, other studies have shown that increasing quantities of virus shedding by infected animals is associated with higher probabilities of transmission (25–27). Thus, the assumption that detection of any viral RNA indicates contagiousness may lead to overestimation of the duration of the infectious period. In absence of data confirming actual transmission, experimentally defined thresholds for virus shedding may represent a quantifiable approach for standardized estimation of the onset and duration of infectiousness.

**Figure 1:**
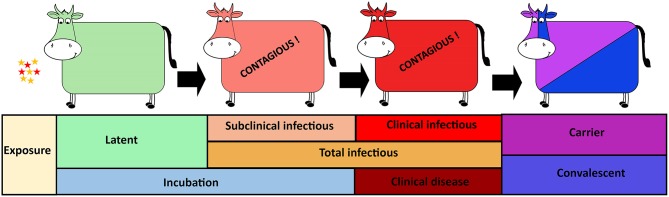
Foot-and-mouth disease virus (FMDV) progression in cattle with distinct phases of infection from exposure to carrier or convalescent. The FMDV exposed cattle sequentially transverse latent, subclinical infectious, and clinical infectious phases. The incubation phase includes the latent- and subclinical infectious phases whereas the total infectious phase includes the subclinical infectious- and clinical infectious phases.

The purpose of the current study was to provide improved estimates of the durations of specific phases of FMDV infection in cattle (latent, incubation, subclinical infectious, clinical infectious, and total infectious periods; Figure 1). Aggregate (pan-serotypic) and stratified parameters for three serotypes of FMDV in cattle were generated considering different indicators of infectiousness as defined by virus shedding (with or without thresholds) in nasal secretions. Finally, the influence of specific components of experimental design upon disease phase durations was investigated.

## Materials and Methods

### Data

Analyses were based on data derived from experimental studies (Supplementary Table S1) investigating FMDV pathogenesis and infection dynamics in cattle, conducted at Plum Island Animal Disease Center (PIADC), New York, USA between 2011 and 2016 (20, 22–24, 28, 29). These experiments were originally designed for varying objectives but followed a similar overarching study design and sampling approach. In brief, cattle were infected with one out of five different FMDV strains (O/SKR/2010, O_1_Manisa, O_1_Campos, A_24_ Cruzeiro, and Asia_1_ Shamir) through either direct inoculation or through contact exposure to infected (inoculated) cattle or pigs. Inoculation systems used were intra-dermal lingual (IDL), intra-nasopharyngeal (INP) (24), or aerosol inoculation (22). While IDL inoculation consists of direct injection of virus inoculum into the epithelium of the tongue, the INP and aerosol inoculation systems have been developed as “simulated natural” (SN) systems that utilize the most likely natural routes of virus exposure. Additionally, direct contact exposure, which was utilized in a number of experiments, represents natural exposure consisting of time-limited co-habitation of experimental animals with either cows or pigs which had been inoculated with FMDV at 24–48 h before the start of the exposure period (20, 24). Across all experiments, 38 cattle were exposed through contact and 63 cattle were inoculated [total cattle (*n*) = 101]. Within contact-exposed cattle, 8 cattle were infected through exposure to FMDV-infected cattle (serotype O: 4 cattle, serotype A: 2 cattle, and serotype Asia1: 2 cattle) and 30 cattle were infected through exposure to FMDV-infected pigs (serotype O: 8 cattle, serotype A: 18 cattle, and serotype Asia1: 4 cattle). Within the inoculated cattle group, six cattle were directly infected via IDL inoculation (serotype O: 2 cattle, serotype A: 2 cattle, and serotype Asia1: 2 cattle), whereas 57 cattle were infected through simulated natural systems (serotype O: 14 cattle, serotype A: 39 cattle, and serotype Asia1: 4 cattle); [intra-nasopharyngeal (INP; *n* = 46) or aerosol inoculation (*n* = 11)].

In all experiments, cattle were monitored through pre-determined durations after infection. A subset of animals was euthanized during the early phase of infection for analysis of FMDV distribution in tissues (not described herein) and were therefore not monitored through to convalescence. Data used for this current investigation consisted of daily to weekly measurements ranging from 0 to 35 days post infection (dpi). FMDV infection dynamics were assessed by monitoring development of characteristic FMD lesions (lesion score) (29), as well as through quantitative measurements of FMDV RNA in nasal swab samples. Nasal secretions extracted from the swabs were processed and analyzed by quantitative RT-PCR as previously described (22). The cycle threshold values of the qRT-PCR assay were converted to FMDV genome copy numbers (GCN)/ml using an equation derived from similar analysis of a dilution series of *in-vitro* derived, strain-specific FMDV cDNA of known concentrations, and FMDV quantities were subsequently expressed as log10 GCN/ml.

### Definition of FMD Phase Durations

The data obtained from the experimental studies were used to estimate the durations of distinct phases of FMDV infection in cattle. Specifically, the presence and quantity (threshold) of FMDV RNA in nasal secretions were used to estimate the onset and decline of infectiousness, thereby defining the duration of the latent period. Similarly, the duration of the incubation period and the onset of the clinical phase were estimated based upon the appearance of FMD lesions. Additionally, combined measures of FMDV shedding and occurrence of FMD lesions were used to further divide the infectious period into subclinical and clinical compartments (Figure 1). All animals included in the analyses were determined to be infected after exposure to FMDV, regardless of the exposure method. The analyses presented herein were based on data from non-vaccinated cattle only, and all animals included developed visible FMD lesions.

Two different approaches were utilized in order to define the onset and end of infectiousness based upon the amount of FMDV shedding in nasal secretions. In the “threshold shedding approach,” cattle were considered infectious when the FMDV RNA shedding was >3.92 log10 GCN/ml in nasal secretions (20, 24). This assumption was based on documented transmission having occurred from a subset of animals with this level of shedding at 24 h post inoculation (hpi). In those cattle, the mean (95% confidence interval) FMDV RNA shedding at 24 hpi was 4.16 (3.92–4.40) log10 GCN/ml. The lower limit of the 95% confidence interval (3.92 log10 GCN/ml) was thereby considered as the minimum quantity of FMDV RNA shedding required for infectiousness. It was not experimentally confirmed that cattle shedding less than this threshold were not infectious. For this approach, the end of infectiousness was defined as the first day after the onset of clinical signs when FMDV shedding declined below the threshold of 3.92 log10 GCN/ml. Additionally, a “non-threshold approach” was similarly applied to define the onset of infectiousness simply requiring that FMDV RNA shedding was >0 log10 GCN/ml in nasal swab samples. This approach is most consistent with previous studies which assumed that any detection of FMDV RNA in clinical samples indicates infectivity. Similarly, for this second scenario, the first day after the onset of clinical signs when the animal had 0 log10 GCN/ml of FMDV RNA shedding was considered as the end of infectiousness.

Since the animals were monitored for variable durations of time, the number of days during which an animal was in a particular disease phase may not have been documented in full. To account for this, some of the measurements were censored for some animals. For example, if the animal was in the clinical infectious phase but was euthanized prior to the estimated end of infectiousness, the number of days that contributed to the clinical infectious phase were documented and counted as right censored for the clinical infectious period. A similar approach was applied for all phases of infection.

### Statistical Analysis

The durations of distinct phases of FMDV infection were estimated for individual cattle using the two approaches of FMDV shedding described above. An Accelerated Failure Time (AFT) model was employed using SAS 9.4 (Cary, NC, USA) to assess the impact of sets of predictor variables on the disease phase durations. AFT is a parametric survival regression model that has been employed previously for similar data, which aims to measure time to event in the presence of data censoring (21, 30). The AFT model assumes that the baseline hazard function approximates the Weibull distribution in which the hazard function is monotonic; i.e., it increases when the shape parameter (p) is >1 and decreases when the p is <1 (30). An AFT model with Weibull distribution was fitted separately for each of the disease phase durations (incubation, latent, subclinical infectious, clinical infectious, and total infectious) for both thresholds for infectiousness. The AFT model provides a “time ratio (TR)” estimate for a predictor, which estimates the relative amount of time to failure due to one predictor variable compared to another predictor variable included in the model (Table 1). While selecting the model predictors, the predictors with Variance Inflation Factor (VIF) >15 were excluded to avoid multicollinearity issues (30). The virus strains O/SKR/2010, O_1_Manisa, and O_1_Campos were combined into one category, serotype O. The FMDV strains Asia1 Shamir, and A_24_ Cruzeiro represented serotypes Asia1 and A, respectively.

**Table 1:** Predictors included in the Accelerated Failure Time (AFT) models for the estimation of length of various phases of foot-and-mouth disease infection in cattle.

**Predictors**	**Categories**	**Descriptions**
1. Virus strains	O/SKR/2010	The experiments were conducted using five virus strains.
	O_1_Manisa (O_1_M)	
	O_1_Campos (O_1_C)	
	Asia1 Shamir	
	A_24_ Cruzeiro (reference)	
2. Virus serotypes	O	The strains O/SKR/2010, O_1_C, and O_1_M were combined to represent the serotype O. Serotype Asia1 and A were represented by Asia1 Shamir and A_24_ Cruzeiro, respectively.
	Asia1	
	A (reference)	
3. Exposure type	Contact	The experimental cattle were exposed with the virus through direct inoculation or direct contact with inoculated cattle or pigs.
	Inoculated (reference)	
4. Exposure method 1 (em1)	Inoculated	All inoculated cattle were put together. The contact-exposed cattle were categorized in two groups: CTC (cattle to cattle transmission) and PTC (pig to cattle transmission).
	CTC	
	PTC (reference)	
5. Exposure method 2 (em2)	Contact	All contact-exposed cattle were put together. The inoculated cattle were categorized as exposure by either IDL (Intra-dermal lingual) or SN (Simulated natural).
	IDL	
	SN (reference)	
6. Exposure method 3 (em3)	CTC	All the exposure methods were categorized separately to assess the role of individual exposure methods.
	PTC	
	IDL	
	SN (reference)	
7. rRT-PCR test kit used	Taq CCC	Two types of rRT-PCR test kits were employed for the estimation of FMDV RNA shedding from the nasal swab samples.
	Ag RCR (reference)	

Since the experimental cattle were exposed either through contact or inoculation, and each exposure method was associated with two factors, we constructed our exposure method variable as described below. Method 1 consisted of all of the inoculated cattle being combined together and the contact-exposed cattle being put into two categories: (a) cattle to cattle transmission (CTC) and (b) pig to cattle transmission (PTC). In CTC and PTC categories, the donor animal species were cattle and pig, respectively. For method 2, the contact-exposed cattle were combined together, but the inoculated cattle were categorized into two groups: (a) intra-dermal lingual (IDL) inoculation and (b) simulated natural (SN) inoculation systems, which included intra-nasopharyngeal (INP) and aerosol inoculation systems. For method 3, each of the exposure methods (CTC, PTC, IDL, and SN) represented one category in the exposure method variable included in the models.

Various AFT models were built for each of the FMDV phase durations through a forward-stepwise procedure considering virus strains, virus serotypes (individual serotypes and pan-serotype), exposure type, exposure methods, and the rRT-PCR test kit used in diagnostics. The pan-serotype level estimation was the weighted average of all serotypes. The category with the highest sample size was selected as a reference group in the model. All possible combinations of predictors were fit, and the most biologically plausible model (based on consensus among the authors) with the lowest AIC (Akaike's Information Criterion) was selected for the respective disease phase durations. To allow for pair-wise comparisons of the effect of predictor categories on phase duration, Tukey adjustments were used, and the reported *p*-values are from these pair-wise comparisons. The models were further tested for confounders and first order interactions among the predictors. The regression coefficient, 95% confidence interval, *p*-value, and Weibull shape parameters were presented for the best fit models. The survival time ratio (TR) was estimated as the exponential of the regression coefficient (β) of the variables.

The estimated disease phase durations derived from the use of a defined threshold of FMDV shedding as a proxy for contagiousness were compared to the estimates resulting from the assumption that any viral RNA detection indicated infectiousness. The purpose was to test the hypothesis that application of a threshold value for contagiousness would affect the estimates of durations of disease phases. To do so, AFT models similar to those described above were fit, with the inclusion of a binary indicator variable, indicating whether the disease phase duration was measured under the “threshold” assumption or the “non-threshold” assumption. To test if there were statistically significant differences in phase durations between the two approaches, a Wald test for the significance of the binary indicator variable in the AFT model was performed.

The probability distribution functions were fit for each of the FMDV phase durations obtained from the animal-level and model-predicted FMDV phase duration data. The commonly used probability distribution functions in FMD simulation models were selected for the distribution fit using @Risk 7.5 (Palisade, New York, USA). For the continuous data, Pert, Gamma, Inverse Gaussian, Logistic, Normal, Weibull, and Lognormal distributions were considered, and for the discrete data Binomial, Negative Binomial, Geometric, and Poisson distributions were considered using one of two goodness of fit tests (Anderson-Darling for continuous data and Chi-square tests for discrete data). Using the maximum likelihood estimates, 1,000 iterations were performed to fit the distribution for each of the phase durations. A significance level of 0.05 was used to assess model fit.

## Results

The durations of distinct phases of FMDV infection were estimated for three serotypes (O, Asia_1_, and A) based on data from 101 total cattle. However, due to variations in design and duration of the experimental studies, the total number of cattle used for estimation of particular phase durations differed. For all of the FMD infection phases (incubation, latent, subclinical infectious, clinical infectious, and total infectious), the best fit model included virus serotype and exposure method 3, which had four categories, one for each of the exposure methods (CTC, PTC, IDL, and SN; see Table 1). “rRT-PCR test kit used” and virus strain were not significant model predictors and were not included in any model in the final model set. For each of the FMD phase durations, the animal-level descriptive findings have been followed by the model-predicted outcomes, stratified by serotype and exposure method, with multiple group comparisons. Outcomes are compared for each of the disease phases assuming infectiousness was determined by threshold-defined or non-threshold-defined levels of shedding.

### Incubation Phase Duration

**Figure 2:**
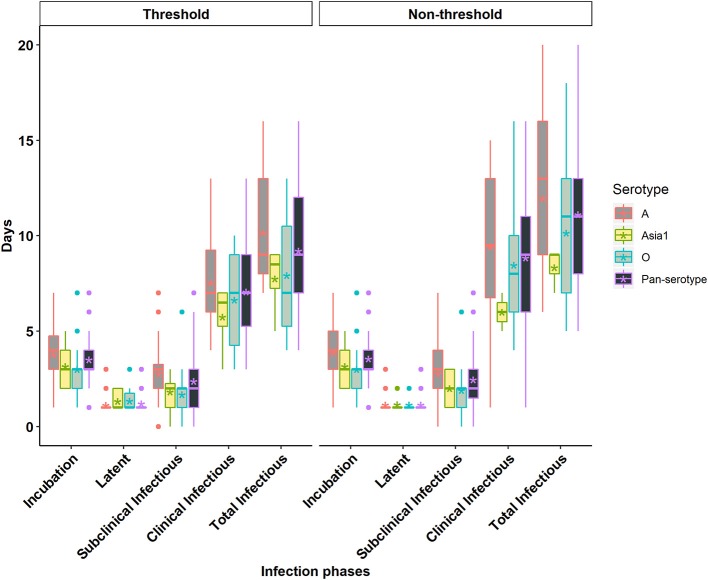
Box plots of the incubation phase, latent phase, subclinical infectious phase, clinical infectious phase, and total infectious phase durations in cattle due to exposure to FMDV serotype A, Asia1, O, and pan-serotype for the threshold-defined and non-threshold approaches for defining infectiousness. The middle, lower, and upper line of the box represents the median, 25th and 75th percentile. The whiskers represent 1.5 times of the interquartile range. The asterisks represent the mean and dots are the outliers detected by the analytic tool.

**Figure 3:**
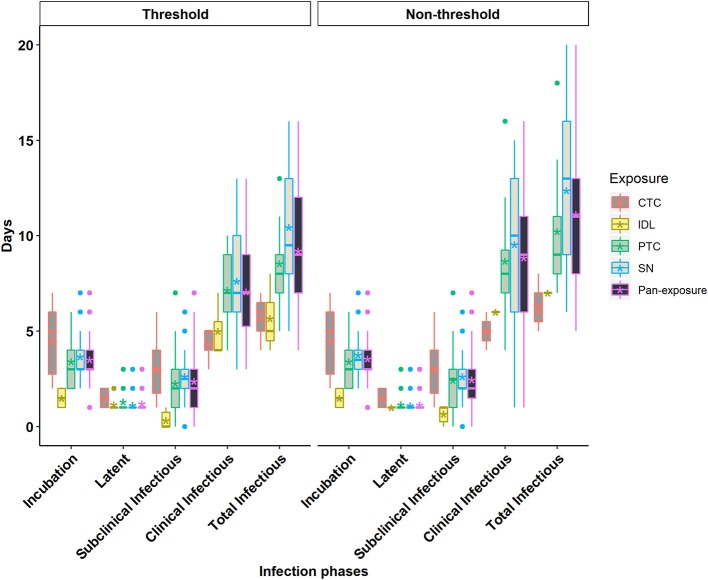
Box plots of the incubation phase, latent phase, subclinical infectious phase, clinical infectious phase, and total infectious phase durations in cattle obtained from cattle to cattle (CTC), pig to cattle (PTC), Intradermal lingual (IDL), simulated natural (SN), and pan-exposure methods for the threshold-defined and non-threshold-defined approaches for defining infectiousness. The middle, lower, and upper line of the box represents the median, 25th and 75th percentile. The whiskers represent 1.5 times of the interquartile range. The asterisks represent the mean and dots are the outliers detected by the analytic tool.

The incubation period corresponds to the time from infection to the appearance of clinical signs of disease (Figure 1). The individual animal-level incubation period ranged from 1 to 7 days (Figures 2, 3 and Supplementary Tables S2, S3). Since the incubation duration describes clinical rather than virological parameters, it was not possible to include the comparison between the threshold and non-threshold approaches used to define the infectious period, as will be described in the sections which follow below.

**Figure 4:**
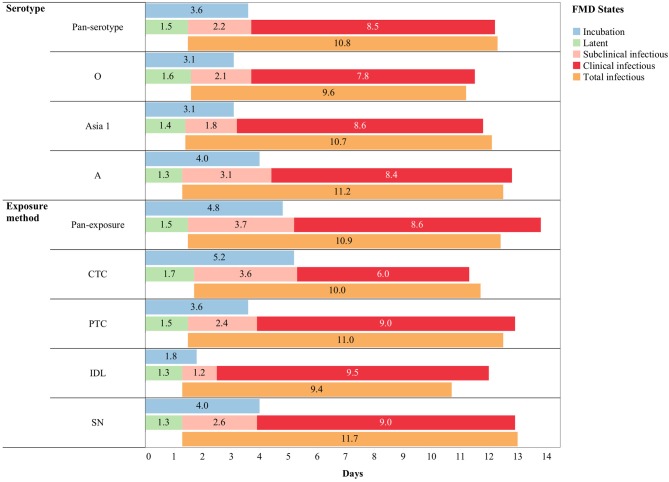
Pan-serotypic and pan-exposure mean durations (in days) of incubation phase (*n* = 88), latent phase (*n* = 101), subclinical infectious phase (*n* = 99), clinical infectious phase (*n* = 89), and total infectious phase (*n* = 99) of foot-and-mouth disease infection (threshold-defined approach only) in cattle.

The mean (95% Cl) incubation duration of pan-serotype FMD was 3.6 days (2.7–4.8; Figure 4). From the AFT model (Table 2), the mean (95% Cl) incubation duration was estimated to be 3.9 days (3.5–4.4) for serotype A (longest) and 3.1 (2.7–3.6) for serotype O (shortest). Compared to serotype A, serotype O (*p* = 0.0062) and Asia1 (*p* = 0.036) resulted in 0.8 times shorter incubation duration, whereas there was no significant difference between serotypes O and Asia1 (*p* = 0.99).

**Table 2:** The best-fit Accelerated Failure Time (AFT) model for the incubation phase duration (same model for both threshold-defined and non-threshold infectiousness) in cattle (Weibull distribution shape parameter = 3.4), n is the number of animals in each category.

**Variables**	**Category**	***n***	**Coefficient (β)**	**95% Cl**	**Time ratio**	***p*-value**
	Intercept		1.51	1.42 to 1.59		<0.0001
Serotype	O	26	−0.21	−0.37 to −0.06	0.8	0.0053
	Asia1	12	−0.22	−0.43 to −0.01	0.8	0.0379
	A	60	0			
Exposure method 3	CTC	8	0.29	0.058 to 0.52	1.3	0.0147
	PTC	29	−0.08	−0.22 to 0.06	0.9	0.2713
	IDL	6	−0.78	−1.06 to −0.5	0.5	<0.0001
	SN	55	0			

Across the exposure methods, the incubation period was 1.3 times longer when cattle were exposed via CTC (*p* = 0.0147), and it was 0.5 times shorter for the IDL exposed cattle (*p* < 0.0001) compared to SN exposure methods (Table 2). Notably, the incubation period was not different between cattle exposed via SN and PTC exposure methods (*p* = 0.6894). CTC exposure resulted in 3 times longer incubation duration than IDL (*p* < 0.0001) and 1.4 times longer than PTC (*p* = 0.0119). Similarly, the PTC exposure method resulted in 2 times longer incubation duration compared to IDL (*p* < 0.0001). The duration of the incubation period was longest (6 days) for serotype A (exposure method: CTC) and shortest (1.7 days) for serotype O (exposure method: IDL). The model-predicted incubation duration is detailed in Tables 3A,B; the distributions fit for the incubation durations obtained from animal-level and model-predicted data are summarized in Supplementary Table S4.

**Table 3A:** The mean (95% Cl) length of various FMD infection phases obtained from the best-fit Accelerated Failure Time (AFT) model.

**FMD phases**	**Serotype**			**Exposure method**			
	**O**	**Asia1**	**A**	**CTC**	**PTC**	**IDL**	**SN**
**THRESHOLD-DEFINED APPROACH**							
Incubation	3.1 (2.7–3.6)	3.1 (2.6–3.7)	4 (3.5–4.4)	5.2 (4.2–6.4)	3.6 (3.2–4.1)	1.8 (1.4–2.3)	4 (3.5–4.3)
Latent	1.6 (1.4–2)	1.4 (1.2–1.8)	1.3 (1.1–1.5)	1.7 (1.3–2.2)	1.5 (1.3–1.8)	1.3 (1–1.8)	1.3 (1.2–1.5)
Subclinical infectious	2.1 (1.6–2.6)	1.8 (1.4–2.4)	3.1 (2.5–3.8)	3.6 (2.7–5)	2.4 (2–2.8)	1.2 (0.7–2.1)	2.6 (2.2–3)
Clinical infectious	7.8 (6.6–9.1)	8.6 (6.7–11)	8.4 (7.4–9.6)	6 (4.5–8)	9 (7.7–10.5)	9.5 (7.1–12.6)	9 (8–10.2)
Total infectious	9.6 (8.3–11)	10.7 (8.5–13.4)	11.2 (10–12.6)	10 (7.7–13)	11 (9.5–12.5)	9.4 (7.3–12.1)	11.7 (10.4–13.1)
**NON-THRESHOLD APPROACH**							
Incubation	3.2 (2.8–3.6)	3.1 (2.7–3.7)	4 (3.5–4.4)	5.2 (4.3–6.4)	3.6 (3.2–4.1)	1.8 (1.4–2.3)	4 (3.5–4.3)
Latent	1.3 (1.1–1.5)	1.2 (1–1.4)	1.3 (1.2–1.5)	1.7 (1.3–2.1)	1.3 (1.1–1.5)	1 (0.8–1.3)	1.2 (1.1–1.3)
Subclinical infectious	2.1 (1.8–2.5)	2 (1.6–2.4)	3 (2.5–3.6)	3.7 (2.8–4.8)	2.6 (2.2–3.0)	1.1 (0.8–1.7)	2.7 (2.3–3)
Clinical infectious	9.9 (8–12.4)	10.1 (7.1–14.3)	10.7 (8.7–13.3)	6.6 (4.7–9.2)	10.8 (9–13.1)	13.8 (7.8–24.6)	11.1 (9.3–13.3)
Total infectious	12.2 (10–15)	12.5 (9.2–17.1)	13.6 (11.3–16.5)	10.4 (7.7–14)	12.9 (10.9–15.2)	14.1 (8.4–23.7)	14.1 (12–16.5)

*The outcomes are stratified by serotypes and exposure methods for both threshold approaches for definition of infectiousness*.

**Table 3B:** The mean (95% Cl) length of various FMD infection phases obtained from the best-fit Accelerated Failure Time (AFT) model.

**FMD phases**	**Pan-serotype**	**Exposure methods**			
		**CTC**	**PTC**	**IDL**	**SN**
**THRESHOLD-DEFINED APPROACH**					
1. Incubation (*n* = 88)	3.6 (2.7–4.8)	5.5 (4.4–6.8)	3.8 (3.4–4.3)	1.8 (1.4–2.3)	4.2 (3.8–4.6)
2. Latent (*n* = 101)	1.5 (1.1–2.1)	1.6 (1.2–2.1)	1.5 (1.3–1.7)	1.2 (0.9–1.7)	1.3 (1.2–1.4)
3. Subclinical infectious (*n* = 99)	2.2 (1.5–3.5)	4.1 (3.0–5.6)	2.8 (2.3–3.2)	1.2 (0.7–2.2)	3.0 (2.7–3.4)
4. Clinical infectious (*n* = 89)	8.5 (6.2–11.6)	6.1 (4.6–8.1)	9.1 (7.8–10.5)	9.6 (7.3–12.6)	8.9 (8.1–9.9)
5. Total infectious (*n* = 99)	10.8 (8.2–14.2)	10.2 (7.9–13.2)	11.1 (9.8–12.6)	9.6 (7.5–12.4)	11.9 (10.9–13)
**NON-THRESHOLD APPROACH**					
1. Incubation (*n* = 88)	3.6 (2.7–4.8)	5.4 (4.4–6.7)	3.8 (3.4–4.3)	1.8 (1.4–2.3)	4.2 (3.8–4.5)
2. Latent (*n* = 101)	1.2 (0.9–1.6)	1.6 (1.3–2.1)	1.3 (1.2–1.5)	1.0 (0.8–1.3)	1.2 (1.1–1.4)
3. Subclinical infectious (*n* = 101)	2.4 (1.6–3.5)	4 (3–5.4)	2.9 (2.5–3.3)	1.2 (0.8–1.7)	3.0 (2.7–3.4)
4. Clinical infectious (*n* = 89)	10.8 (6.8–17)	6.7(4.7–9.4)	11.1 (9.2–13.4)	14.0 (7.9–25)	11.4 (10.1–12.9)
5. Total infectious (*n* = 101)	13.4 (9.0–20.0)	10.6 (7.9–14.4)	13.2 (11.3–15.5)	14.4 (8.6–24.2)	14.5 (12.9–16.2)

*The outcomes are non-stratified for serotypes (pan-serotype) and stratified only for exposure methods for both approaches of definition of infectiousness, n is the number of animals in each category used to estimate the state durations at pan-serotype level*.

### Latent Phase Duration

The latent period represents the time elapsed from infection until the onset of infectiousness, as defined by either of the two approaches for defining infectiousness using FMDV shedding in nasal secretions (Figure 1). The individual animal-level latent phase ranged from 1 day to 3 days (Figures 2, 3 and Supplementary Tables S2, S3). The use of threshold or non-threshold approaches to define the onset of infectiousness did not result in any significant difference in the duration of latency at the individual animal level (*p* = 0.08).

The mean (95% Cl) latent duration of pan-serotype FMD was 1.5 days (1.1–2.1) based on the threshold approach (Figure 4). The mean (95% Cl) latent duration was estimated to be 1.3 days (1.1–1.5) for serotype A (shortest), 1.6 days (1.4–2) for serotype O (longest), and 1.4 days (1.2–1.8) for serotype Asia_1_ (Table 3A). From the best-fit model (Table 4), the latent duration was not different between the serotypes when using the non-threshold approach to define infectiousness. However, it was 1.2 times longer for animals infected by FMDV serotype O (*p* = 0.0184) when the threshold was used.

**Table 4:** The best-fit Accelerated Failure Time (AFT) model for the latent phase duration (threshold-defined and non-threshold approaches) in cattle (Weibull distribution shape parameter = 2.60 for threshold and 2.83 for non-threshold), *n* is the number of animals in each category.

**Variables**	**Category**	***n***	**Coefficient (β)**	**95% Cl**	**Time ratio**	***p-*value**
**MODEL FOR THRESHOLD-DEFINED APPROACH**						
	Intercept		0.18	0.07–0.29		0.0017
Serotype	O	28	0.22	0.04–0.39	1.2	0.0184
	Asia1	12	0.09	−0.2–0.36	1.1	0.5046
	A	61	0			
Exposure method 3	CTC	8	0.24	−0.06–0.54	1.3	0.118
	PTC	30	0.15	−0.03–0.32	1.2	0.1016
	IDL	6	−0.04	−0.40–0.32	0.96	0.831
	SN	57	0			
**MODEL FOR NON-THRESHOLD APPROACH**						
	Intercept		0.23	0.12–0.33		<0.0001
Serotype	O	28	−0.05	−0.20–0.11	0.95	0.5457
	Asia1	12	−0.13	−0.37–0.11	0.88	0.2885
	A	61	0			
Exposure method 3	CTC	8	0.34	0.05–0.62	1.4	0.0202
	PTC	30	0.09	−0.06–0.25	1.1	0.2382
	IDL	6	−0.16	−0.47–0.15	0.85	0.3017
	SN	57	0			

The exposure method was not an influential factor for the duration of the latent phase estimated using the threshold approach, but when using the non-threshold approach, CTC exposure resulted in 1.4 times longer latent duration than the simulated natural exposure methods (*p* = 0.0202) and 1.6 times longer than the IDL exposure method (*p* = 0.0453). Using cattle as donors for contact exposure (CTC) resulted in 1.3 times longer latent duration than when pigs were used as donors (PTC) (*p* = 0.09). Among the inoculated animals, the duration of the latent period was slightly longer for the SN group than the IDL group (but not significantly different) in both approaches for defining infectiousness. The model-predicted latent duration of FMD is summarized in Tables 3A,B. The distributions fit for the animal-level and model-predicted latent duration are summarized in Supplementary Table S4 (threshold approach) and Supplementary Table S5 (non-threshold approach).

### Subclinical Infectious Phase Duration

The subclinical infectious period was defined as the duration from the onset of infectiousness (defined separately by either of the two approaches for defining infectiousness) until the appearance of clinical signs of disease at the end of the incubation phase (Figure 1). The animal-level subclinical infectious period ranged from 0 to 7 days (Figures 2, 3 and Supplementary Tables S2, S3). The animal-level mean subclinical infectious duration was not significantly different (*p* > 0.05) between the approaches used to define infectiousness.

The mean (95% Cl) subclinical infectious duration of pan-serotype FMD was 2.2 days (1.5–3.5) for the threshold-defined approach (Figure 4). From the AFT model (Table 5), the mean (95% CI) subclinical infectious duration was estimated to be 3.1 days (2.5–3.8) for serotype A, 1.8 days (1.4–2.4) for serotype Asia1, and 2.1 days (1.6–2.6) for serotype O. The duration was not significantly different between serotypes Asia1 and O (*p* = 0.7303). The subclinical infectious duration for cattle infected with FMDV serotype O and Asia1 was 0.7 (*p* = 0.0008 in threshold and 0.0012 in non-threshold) and 0.6 (*p* = 0.0003 in threshold and *p* = 0.001 in non-threshold) times shorter than for animals infected with serotype A, respectively, in both approaches for defining infectiousness.

**Table 5:** The best-fit Accelerated Failure Time (AFT) model for the subclinical infectious phase duration (threshold-defined and non-threshold defined infectiousness) in cattle (Weibull distribution shape parameter = 2.47 for threshold and 2.59 for non-threshold), n is the number of animals in each category.

**Variables**	**Category**	***n***	**Coefficient (β)**	**95% Cl**	**Time ratio**	***p*-value**
**MODEL FOR THRESHOLD-DEFINED APPROACH**						
	Intercept		1.26	1.12 to 1.39		<0.0001
Serotype	O	26	−0.4	−0.63 to −0.17	0.7	0.0008
	Asia1	12	−0.52	−0.79 to −0.24	0.6	0.0003
	A	61	0			
Exposure method 3	CTC	8	0.34	0.02 to 0.67	1.4	0.0397
	PTC	30	−0.09	−0.28 to 0.10	0.9	0.3631
	IDL	6	−0.8	−1.38 to −0.20	0.5	0.0088
	SN	55	0			
**MODEL FOR NON-THRESHOLD APPROACH**						
	Intercept		1.24	1.11 to 1.36		<0.0001
Serotype	O	28	−0.34	−0.55 to −0.14	0.7	0.0012
	Asia1	12	−0.44	−0.69 to −0.17	0.6	0.001
	A	61	0		1.0	
Exposure method 3	CTC	8	0.32	0.01 to 0.63	1.4	0.0418
	PTC	30	−0.03	−0.22 to 0.15	1.0	0.7346
	IDL	6	−0.84	−1.26 to −0.42	0.4	<0.0001
	SN	57	0			

Across the exposure methods, CTC-exposed cattle had the longest subclinical infectious duration, whereas IDL inoculation resulted in the shortest duration in both approaches of defining infectiousness (Table 5). Among the CTC-exposed cattle, it was 1.5 times and 3.1 times longer compared to PTC (*p* = 0.0618) and IDL (*p* = 0.0025), respectively. The duration of subclinical infectiousness was 2 times longer when the exposure was via PTC compared to IDL (*p* = 0.1014). However, it was not significantly different between the cattle exposed via PTC and SN (*p* = 0.79). The subclinical infectious period was longest (5 days) for serotype A (exposure method: CTC) and shortest (1 day) for the serotype Asia1 (exposure method: IDL). The model-predicted subclinical infectious duration of FMD is in Tables 3A,B. The distributions fit for the animal-level and model-predicted subclinical infectious duration data are summarized in Supplementary Table S4 (threshold) and Supplementary Table S5 (non-threshold).

### Clinical Infectious Phase Duration

The clinical infectious period was defined as the time elapsed from the onset of clinical signs until the end of infectiousness as defined by either of the two approaches for defining infectiousness (Figure 1). The animal-level clinical infectious period ranged from 1 to 16 days when defining infectivity by the non-threshold approach, and 3 to 13 days when using the threshold-defined approach (Figures 2, 3 and Supplementary Tables S2, S3). The animal-level mean clinical infectious period was 1.25 times longer when infectiousness was defined by the non-threshold (*p* = 0.005).

The mean (95% Cl) clinical infectious duration of pan-serotype FMD was 8.5 days (6.2–11.6) for the threshold approach (Figure 4). From the best fit model (Table 6), the mean (95% Cl) clinical infectious duration in cattle was estimated to be 8.4 days (7.4–9.6) for serotype A, 7.8 days (6.6–9.1) for serotype O, and 8.6 days (6.7–11.0) for serotype Asia1. The model showed that the clinical infectious duration was not significantly different based on serotypes, however, Asia1 for the threshold approach (8.6 days) and serotype A for non-threshold approach (10.7 days) had the longest clinical infectious duration. In both approaches for defining infectiousness, serotype O had the shortest (7.8 days for high threshold and 9.9 days for low threshold) clinical infectious duration.

**Table 6:** The best-fit Accelerated Failure Time (AFT) model for the clinical infectious phase duration (threshold-defined and non-threshold defined approaches) in cattle (Weibull distribution shape parameter = 4.08 for threshold and 3.46 for non-threshold), n is the number of animals in each category.

**Variables**	**Category**	***n***	**Coefficient (β)**	**95% Cl**	**Time ratio**	***p*-value**
**MODEL FOR THRESHOLD APPROACH**						
	Intercept		2.22	2.1–2.3		<0.0001
Serotype	O	22	−0.09	−0.25 to 0.08	0.9	0.3302
	Asia1	12	0.02	−0.25 to 0.29	1.0	0.9096
	A	55	0			
Exposure method 3	CTC	8	−0.4	−0.7 to −0.09	0.7	0.0112
	PTC	29	0.006	−0.17 to 0.18	1.0	0.9508
	IDL	6	0.06	−0.24 to 0.35	1.1	0.7115
	SN	46	0			
**MODEL FOR NON-THRESHOLD APPROACH**						
	Intercept		2.46	2.33 to 2.57		<0.0001
Serotype	O	22	−0.08	−0.31 to 0.16	0.9	0.5207
	Asia1	12	−0.06	−0.44 to 0.31	0.9	0.7397
	A	55	0			
Exposure method 3	CTC	8	−0.52	−0.90 to −0.15	0.6	0.0065
	PTC	29	−0.03	−0.26 to 0.21	1.0	0.8282
	IDL	6	0.22	−0.38 to 0.81	1.2	0.4778
	SN	46	0			

Some exposure methods highly influenced the estimates of the clinical infectious duration. The cattle exposed by CTC had 0.7 times shorter clinical infectious duration than the cattle exposed through SN methods (*p* = 0.0112; Table 6). Compared to PTC and IDL exposure methods, the clinical infectious period was shorter for CTC-exposed cattle by 0.67 times (*p* = 0.055) and 0.63 times (*p* = 0.117), respectively. Notably, the clinical infectious durations were approximately the same when the cattle were exposed by either PTC or IDL, PTC or SN, and IDL or SN. Overall, the longest (13.8 days) clinical infectious duration was estimated for IDL exposed cattle, whereas it was shortest (6.6 days) for cattle exposed via the CTC method. A similar trend was found in the models obtained from the data with the non-threshold. The model-predicted clinical infectious duration of FMD is in Tables 3A,B. The distributions fit for the clinical infectious duration obtained from animal-level and model-predicted data are summarized in Supplementary Table S4 (threshold) and Supplementary Table S5 (non-threshold).

### Total Infectious Phase Duration

The total infectious duration is comprised of the combined subclinical- and clinical infectious periods and was thus delineated by either the non-threshold or threshold-defined FMDV shedding approaches. The animal-level total infectious duration ranged from 5 to 20 days when based on the non-threshold and 4 to 16 days when using the threshold-defined approach for defining infectiousness (Figures 2, 3 and Supplementary Tables S2, S3). The animal-level mean total infectious duration was 1.2 times longer for non-threshold category (*p* = 0.001).

The mean (95% Cl) total infectious duration of FMDV pan-serotype was 10.8 days (8.2–14.2) when using the threshold-defined approach of infectiousness (Figure 4). From the AFT model (Table 7), the mean (95% Cl) total infectious duration was 11.2 days (9.9–12.6) for serotype A, 9.6 days (8.3–11.0) for serotype O, and 10.7 days (8.5–13.4) for serotype Asia1. Serotype O resulted in significantly shorter (*p* = 0.0474) total infectious duration compared to serotype A; however, it was not different between Asia1 & O and Asia1 & A (*p* > 0.05). Overall, the total infectious duration was longest for serotype A for both approaches to defining infectiousness (11.2 days for threshold and 13.6 days for non-threshold).

**Table 7:** The best-fit Accelerated Failure Time (AFT) model for the total infectious phase duration (threshold-defined and non-threshold approach) in cattle (Weibull distribution shape parameter = 4.49 for threshold and 3.86 for non-threshold), n is the number of animals in each category.

**Variables**	**Category**	***n***	**Coefficient (β)**	**95% Cl**	**Time ratio**	***p*-value**
**MODEL FOR THRESHOLD-DEFINED APPROACH**						
	Intercept		2.52	2.42 to 2.62		<0.0001
Serotype	O	26	−0.15	−0.30 to −0.002	0.9	0.0474
	Asia1	12	−0.05	−0.29 to 0.20	1.0	0.7177
	A	61	0			
Exposure method 3	CTC	8	−0.16	−0.43 to 0.12	0.9	0.2622
	PTC	30	−0.07	−0.23 to 0.08	0.9	0.3506
	IDL	6	−0.22	−0.5 to 0.05	0.8	0.114
	SN	55	0			
**MODEL FOR NON-THRESHOLD APPROACH**						
	Intercept		2.71	2.6 to 2.82		<0.0001
Serotype	O	28	−0.11	−0.31 to 0.09	0.90	0.2916
	Asia1	12	−0.09	−0.42 to 0.25	0.92	0.614
	A	61	0			
Exposure method 3	CTC	8	−0.30	−0.64 to 0.03	0.74	0.0764
	PTC	30	−0.09	−0.29 to 0.12	0.92	0.4018
	IDL	6	0.004	−0.54 to 0.54	1.00	0.9888
	SN	57	0			

The total infectious duration was not significantly different across the exposure methods. However, the CTC exposure method resulted in 1 day and 1.7 days shorter total infectious duration than the PTC and SN, respectively (Table 7). In the case of the non-threshold, the CTC exposed cattle had 2.5 days shorter total infectious duration than PTC and 3.7 days shorter than SN or IDL. The model-predicted total infectious duration of FMD is in Tables 3A,B. The distributions fit for the total infectious duration obtained from animal-level and model-predicted data have been summarized in Supplementary Table S4 (threshold) and Supplementary Table S5 (non-threshold).

## Discussion

The current study was carried out to estimate the duration of distinct phases of early FMD infection (incubation, latent, subclinical infectious, clinical infectious, and total infectious) in cattle including stratification by three FMDV serotypes and 4 routes of exposure to virus. Although the FMDV carrier phase is critically important for regulatory aspects of outbreak control and recovery, transmission from persistently infected cattle is generally believed to be exceedingly low or negligible (31–34); on this basis, parameterization of the carrier state has not been included in this study. Recent knowledge of FMDV pathogenesis and infection dynamics in cattle were incorporated into these analyses and the intricate relationship of the FMD virus type, experimental exposure methods, amount of virus shedding in nasal secretions, and rRT-PCR systems used for FMDV detection were considered in the AFT models. Our findings demonstrated that the progression of FMDV infection, defined by the duration of distinct phases of disease, was substantially affected by virus serotypes, experimental exposure methods, as well as the application of threshold-defined level of viral shedding used to define the onset and end of infectiousness.

This study examined threshold-defined and non-threshold detection of viral shedding to define the onset and end of infectiousness. The non-threshold approach was consistent with the assumption that any detection of viral RNA indicates viral shedding and infectiousness, as has been previously published. However, recent work has indicated that transmission is unlikely to occur with low levels of viral shedding (26, 27). This concept was supported in the current work, by demonstration that the use of non-threshold-limited detection of any viral RNA as a proxy for transmission led to an increased duration of the infectious period. Furthermore, our results suggest that estimates obtained using any shedding as a proxy for infectiousness may be very sensitive to changes associated with the experimental system used to infect the animals. For the purposes of these analyses, a new proxy was utilized based upon data from an experimental transmission study, which found that shedding of 3.92 log10 GCN/ml in nasal secretions was associated with successful transmission events when cattle were used as virus donors (20, 24). Use of this threshold resulted in a shortened infectious period and less sensitivity of the latent period duration to exposure methods. The probability of disease transmission is likely dependent on multiple factors, and the use of a threshold of virus shedding to indicate contagiousness is a simplification of biological processes. However, given the information available in the current data set, combined with evidence from previous works, we found that the approach of defining infectiousness based on measured quantities of virus in nasal secretions provided more realistic estimates of the infectious period.

Due to lack of available resources in the published literature, we cannot compare our findings from serotype A and Asia1 to other studies. However, the estimated length of latent and total infectious phases for serotype O presented herein were longer than what was previously estimated, whereas the duration of the subclinical infectious and incubation phases were comparable (21). An experiment conducted in the Netherlands reported an incubation period of 1–2 days among dairy cattle, which was shorter than our estimates (35). These differences might be attributable to the type of samples tested and the specifications used to define infectiousness. Specifically, the experiments included in the work by Mardones et al. (21) used detection of FMDV RNA or infectious virus in any sample to define infectiousness, with no application of a threshold quantity for infectiousness. Samples included were blood, nasal swabs, oropharyngeal fluids, secretions from prepuce, rectum or vagina, and excretions such as urine, milk, and semen. Charleston et al. estimated that the subclinical infectious period in cattle lasted 1.5 days when nasal fluid samples were used as a proxy for infectiousness, and was less than a day, when experimentally determined by one-to-one contact transmission trials lasting for 8 h (26). Similar to other recent studies, our findings suggest that the subclinical infectious period in cattle may be substantially longer (19, 35, 36). Given the differences in the duration of the infectious period achieved using these different proxies, identifying the best proxy for the onset of infectiousness should be considered during parameter development. In addition, given recent findings in pigs (25, 27) and cattle (26), it is unlikely that using detection of any viral RNA (no threshold shedding) is an appropriate manner of defining infectiousness. Rather, detection of viral RNA in an animal represents a subset of detection of infectious virus, which is similarly a subset of conditions of actual contagiousness.

The different FMDV serotypes included in the current investigation also influenced the disease phase durations, even when adjusting for exposure method. However, it must be noted that the virus strains included herein only represent a very limited selection of the vast diversity of viral strains that comprise each of the FMDV serotypes. It is therefore important to note that although significant differences in disease phase durations were associated with the different serotypes studied herein, substantial differences in pathogenesis may also exist amongst virus strains within the same serotype. More specifically, differences within individual serotypes may be greater than differences across serotypes. For these reasons, the pan-serotype parameterization may be recommended for modeling applications that aim to examine the relative merits of different control strategies in the absence of outbreak data, despite the effect of serotype identified in this paper. However, the serotype-specific parameters may also be useful for modelers seeking to evaluate the impact of this variability on recommended control strategies or predicted disease spread, under very well-defined scenarios.

Previous works have suggested that a high FMDV exposure dose will increase the overall probability of infection (37), and may affect the resulting infection dynamics by reducing the duration of latency and incubation periods (25, 27, 38). In the current study, exposure dose was not included in the models due to differences in virus quantification systems across studies (including titration methods, cell types, and units of measurement) which precluded direct comparisons. However, it is likely that the demonstrated effect of experimental exposure system on FMD phase durations seen in the current study is related to associated variations in the resulting exposure dose. Specifically, when adjusting for serotype, the latent, subclinical infectious, and incubation durations were longer and clinical infectious period was shorter among the cattle exposed to donor cattle (i.e., CTC) compared to donor pigs (i.e., PTC). These differences may be attributed to species-defined variation in the quantity and quality of virus shed by the virus donors during the exposure period. During contact exposure, the effective exposure dose will differ between exposure to cattle and pigs. For example, pigs expel a large amount of aerosolized virus (39), and cattle are highly susceptible to FMDV infection through the respiratory route. Subsequently, the effective exposure dose in pig-to-cattle transmission experiments is likely to be higher than when cattle serve as the donor (24, 27). Other factors which cannot be controlled or quantitated during the experimental design such as differences in virus predilection sites and the differences in how pigs and cattle interact may also have affected the effective exposure dose and contact rates, respectively.

Epidemiological parameters derived from experimental studies have limitations when extrapolating to how the pathogen might behave at the population level. Many factors may influence the disease dynamics within individual animals and herds including individual and herd-level immunity, farm and pen-level management practices, the rate and structure of contacts between animals, and herd size and age composition. In contrast to experimental studies, under field conditions, animals may receive highly varying exposure doses due to these factors and experience varying disease progression (40, 41). An additional complication of this, and all FMDV parameterization studies, is the lack of experimental basis for determining the end of the infectious period. As an FMDV-infected animal mounts an increasing immunological response to infection, the amount of infectious virus that is shed may decline, particularly as infectious virus in secretions will be partially neutralized by secreted antibody. As a result, the quantities of viral shedding required for transmission are likely to be higher in later phases of infection, leading to a shorter infectious period overall. The end of the infectious period is a topic which has received very little attention in the scientific literature and further work is needed to improve our understanding of the best proxies to determine the duration of infectiousness.

Despite these limitations, in cases in which a specific outbreak virus is unknown, average (pan-serotype) estimates of disease phase durations are useful for exploring disease spread and control. As the variability observed amongst the strains and serotypes included in this study suggest, once an outbreak has occurred, every effort should be made to update disease-related parameters to best reflect the specific virus and population at risk. Similarly, in endemic countries which may have co-circulating populations of unique viruses, epidemiologic parameters should be developed that better reflect the highly variable ecology of FMDV in those distinct settings (42–44).

## Conclusion

The purpose of this study was to estimate pan-serotypic and serotype-specific FMD phase durations in cattle considering different approaches for defining infectiousness (threshold and non-threshold defined) using virus shedding in nasal secretions as an indicator of infectiousness. We identified several factors related to experimental design which may be taken into account when estimating parameters, including the virus exposure dose, route of exposure or inoculation, and the donor species used for contact exposure. We found that the proxy used to define the onset or end of infectiousness had a significant impact on the estimated length of the infectious period. Similarly, the experimental system used to expose animals to virus had a significant impact on the length of the latent period. Although serotype-specific and route-specific parameterization are useful for modeling applications, pan-serotypic, all-inclusive parameters may be more appropriate for disease spread modeling to support preparedness for an FMD outbreak because of the robustness derived from inclusion of a diverse range of viruses and exposure routes. This work updates previous, more limited efforts to parameterize phases of FMD in cattle. However, additional transmission studies are needed to validate the best proxies for the onset and the end of the infectious period.

## Author Contributions

SY, CS, AD, and JA: conceptualization of research and study design. CS and JA: execution of animal experimetns. SY, MB, KM-T, and LH: data analysis and visualization. SY: primary drafting of the manuscript. CS, AD, and JA: contributions to writing, reviewing, and editing the manuscript.

### Conflict of Interest Statement

The authors declare that the research was conducted in the absence of any commercial or financial relationships that could be construed as a potential conflict of interest.
