# Supplementary material for: Parameterization of the Durations of Phases of Foot-And-Mouth Disease in Cattle
Source: Front Vet Sci. 2019 Aug 9;6:263. doi: 10.3389/fvets.2019.00263 (PMC6696987; doi:10.3389/fvets.2019.00263)
Supplement: Supplementary file 1 [file Table_1.pdf]

Supplementary Material

Parameterization of the Durations of Phases of Foot-And-Mouth Disease in Cattle

Shankar Yadav[1,2,3], Carolina Stenfeldt[1,4], Matt A. Branan[2], Karla I. Moreno-Torres[1,2,3,] Lindsey Holmstrom[2], Amy H. Delgado[2], Jonathan Arzt[1*]

[1] Foreign Animal Disease Research Unit, Plum Island Animal Disease Center, Agricultural Research Service, United States Department of Agriculture, Greenport, NY, USA

[2]Monitoring and Modeling, Center for Epidemiology and Animal Health, Animal and Plant Health Inspection Service, United States Department of Agriculture, Fort Collins, CO, USA

[3]PIADC Research Participation Program, Oak Ridge Institute for Science and Education, Oak Ridge, TN, USA

[4]Department of Veterinary Population Biology, University of Minnesota, St. Paul, MN, USA

*Corresponding author: Jonathan.Arzt@ARS.USDA.GOV

Supplementary Table S1: Details of the experiments included in this study, conducted at the USDA ARS Plum Island Animal Disease Center during 2011-2016 to accomplish various objectives related to the pathogenesis of the foot-and-mouth disease in cattle.

Studies	Virus strains	Exposure routes	Number of cattle	Inoculation dose	Contact challenge time points
(Pacheco et al., 2016b)	O/SKR/2010	Intra-dermal lingual inoculation	2	10^5.3 PFU	NA
		Cattle to cattle contact	4	NA	24 hpi*
		Pig to cattle contact	4		48 hpi
(Pacheco et al., 2016a)	A24 Cruzeiro	Pig to cattle contact	8	NA	48 hpi
		Intradermal lingual inoculation	2	10^4 TCID50	NA
		Intra-nasopharyngeal inoculation	5	10^5 BTID50	
			2	10^7 TCID50	
		Cattle to cattle contact	2	NA	

	Asia1 Shamir	Intra-dermal lingual inoculation	2	10^4 TCID50	NA
		Intra-nasopharyngeal inoculation	2	10^6 BTID50	
			2	10^7 BTID50	
		Cattle to cattle contact	2	NA	24 hpi
		Pig to cattle contact	4	NA	48 hpi
	O1 Manisa	Intra-nasopharyngeal inoculation	3	10^6 BTID50	
		Pig to cattle contact	4	NA	48 hpi
(Stenfeldt et al., 2016)	A24 Cruzeiro	Intra-nasopharyngeal inoculation	20	10^5 BTID50	NA
			2	10^6 BTID50	
(Stenfeldt et al., 2015)	A24 Cruzeiro	Intra-nasopharyngeal inoculation	10	10^5 BTID50	NA
(Stenfeldt et al., 2018b)	A24 Cruzeiro	Pig to cattle contact	10	NA	48 hpi
(Stenfeldt et al., 2018a)	O1Campos	Aerosol inoculation	8	10^7 PFU	
(Pacheco et al., 2013)	O1Campos	Aerosol inoculation	3	10^7 PFU	

*****hpi – hour post inoculation

Supplementary Table S2: Descriptive statistics for the incubation phase, latent phase, subclinical infectious phase, clinical infectious phase and total infectious phase duration due to FMD virus strains and serotypes from non-censored cattle. n is the number of cattle in each of the category.

Serotypes	Strains	Threshold-defined approach		Non-threshold approach	
		n	Mean, median 25th,75th percentile)	n	Mean, median (25th,75th percentile)
**Incubation phase**					
Pan-serotype	All	88	3.5, 3 (3, 4)	89	3.6, 3 (3, 4)
O	All	22	3, 3 (2, 3)	22	3, 3 (2, 3)
	O/SKR/2010	10	3, 3 (2, 3)	10	3, 3 (2, 3)
	O1Manisa	5	2.6, 2 (2, 2)	5	2.6, 2 (2, 2)
	O1Campos	7	3.3, 3 (3, 3.5)	7	3.3, 3 (3, 3.5)
Asia1	Asia1 Shamir	12	3.2, 3 (2, 4)	12	3.2, 3 (2, 4)
A	A24 Cruzeiro	54	3.8, 4 (3, 4.8)	55	3.9, 3.8 (3, 4)
**Latent phase**					

Pan-serotype	All	99	1.2, 1 (1, 1)	101	1.1, 1 (1, 1)
O	All	26	1.4, 1 (1, 2)	28	1.1, 1 (1, 1)
	O/SKR/2010	10	1.4, 1 (1, 1)	10	1.6, 2 (1, 2)
	O1Manisa	7	1.6, 2 (1, 2)	7	1.6, 2 (1, 2)
	O1Campos	9	1, 1 (1, 1)	11	1.1, 1 (1, 1)
Asia1	Asia1 Shamir	12	1.3, 1 (1, 2)	12	1.2, 1 (1, 1)
A	A24 Cruzeiro	61	1, 1 (1, 1)	61	1.1, 1 (1, 1)
**Subclinical infectious phase**					
Pan-serotype	All	90	2.4, 2 (1, 3)	91	2.5, 2 (1.5, 3)
O	All	22	1.7, 2 (1, 2)	23	1.9, 2 (1, 2)
	O/SKR/2010	10	1.6, 1 (1, 2)	10	2.1, 2 (1, 2)
	O1Manisa	5	1, 1 (0, 1)	6	1.2, 1 (0.3, 1.8)
	O1Campos	7	2.3, 2 (2, 2.5)	7	2.3, 2 (2, 2.5)
Asia1	Asia1 Shamir	12	1.8, 2 (1, 2.3)	12	2, 2 (1, 3)
A	A24 Cruzeiro	56	2.8, 3 (2, 3.3)	56	2.8, 3 (2, 4)
**Clinical infectious phase**					
Pan-serotype	All	42	7.1, 7 (5.3, 9)	40	8.6, 9 (6, 11)
O	All	14	6.6, 7 (4.3, 9)	13	8.5, 8 (6, 10)
	O/SKR/2010	9	5.8, 5 (4, 9)	8	8.3, 7 (5.8, 9.8)
	O1Manisa	2	7, 7 (7, 7)	2	6.5, 6.5 (6.3, 6.8)
	O1Campos	3	9, 10 (8.5, 10)	3	10.3, 10 (10,
Asia1	Asia1 Shamir	4	5.8, 6.5 (5.3, 7)	3	6, 6 (5.5, 6.5)
A	A24 Cruzeiro	24	7.5, 7 (6, 9.3)	24	9.4, 9.5 (6.8, 13)
**Total infectious phase**					
Pan-serotype	All	43	9.2, 9 (7, 12)	41	11.1, 11 (8, 13)
O	All	14	7.9, 7 (5.3, 10.5)	13	10.2, 11 (7, 13)
	O/SKR/2010	9	6.9, 6 (5, 9)	8	10, 9.5 (6.8, 11.8)
	O1Manisa	2	7, 7 (7, 7)	2	6.5, 6.5 (6.8,
	O1Campos	3	11.7, 13 (11, 13)	3	13, 13 (13, 13)
Asia1	Asia1 Shamir	4	7.8, 8.5 (7.3, 9)	3	8.3, 9 (8, 9)
A	A24 Cruzeiro	25	10.2, 9 (8, 13)	25	12, 13 (9, 16)

Supplementary Table S3: Descriptive statistics for the incubation phase, latent phase, subclinical infectious phase, clinical infectious phase and total infectious phase durations due to exposure methods from non-censored cattle. n is the number of cattle in each of the category.

Exposure methods	Threshold-defined approach		Non-threshold approach	
	n	Mean, median 25th, 75th percentile)	n	Mean, median (25th, 75th percentile)
**Incubation phase**				
All	88	3.5, 3 (3, 4)	89	3.6, 3 (3, 4)
Cattle to cattle contact	8	4.5, 5 (2.7, 6)	8	4.5, 5 (2.7, 6)
Pig to cattle contact	29	3.4, 3 (2, 4)	29	3.4, 3 (2, 4)
Intra-dermal lingual inoculation	6	1.5, 1.5 (1, 2)	6	1.5, 1.5 (1, 2)
Simulated natural inoculation	45	3.6, 3 (3, 4)	46	3.7, 3.5 (3, 4)
**Latent phase**				
All	99	1.2, 1 (1, 1)	101	1.1, 1 (1, 1)
Cattle to cattle contact	8	1.5, 1.5 (1, 2)	8	1.5, 1.5 (1, 2)
Pig to cattle contact	30	1, 1 (1, 1)	30	1.1, 1 (1, 1)
Intra-dermal lingual inoculation	6	1.2, 1 (1, 1)	6	1, 1 (1, 1)
Simulated natural inoculation	55	1.1, 1(1, 1)	57	1.1, 1 (1, 1)
**Subclinical infectious phase**				
All	90	2.4, 2 (1, 3)	91	2.5, 2 (1.5, 3)
Cattle to cattle contact	8	3, 3 (1.7, 4)	8	3, 3 (1.7, 4)
Pig to cattle contact	30	2.3, 2 (1, 3)	30	2.4, 2.5 (1, 3)
Intra-dermal lingual inoculation	6	0.3, 0 (0, 0)	6	0.7, 1 (0.25, 1)
Simulated natural inoculation	46	2.6, 2.5 (2, 3)	47	2.6, 2 (2, 3)
**Clinical infectious phase**				
All	42	7.1, 7 (5.3, 9)	40	8.6, 9 (6, 11)
Cattle to cattle contact	3	4.3, 5 (4, 5)	3	5, 5 (4.5, 5.5)
Pig to cattle contact	12	7.1, 7 (6, 9)	12	8.6, 8 (7, 9.2)
Intra-dermal lingual inoculation	3	5, 4 (4, 5.5)	1	6
Simulated natural inoculation	24	7.6, 7 (6, 10)	24	9.5, 10 (6, 13)
**Total infectious phase**				
All	43	9.2, 9 (7, 12)	41	11.1, 11 (8, 13)

Cattle to cattle contact	3	5.6, 6 (5, 6.5)	3	6.3, 6 (5.5, 7)
Pig to cattle contact	13	8.5, 8 (7, 9)	13	10.2, 9 (8, 11)
Intra-dermal lingual inoculation	3	5.6, 5 (4.5, 6.5)	1	7
Simulated natural inoculation	24	10.4, 9.5 (8, 13)	24	12.4, 13 (9, 16)

Supplementary Table S4. The distribution fit for the FMD phase durations obtained from the animal-level data and best-fit AFT model data for threshold-defined approach. The animal-level data are from the non-censored animals only.

Serotypes	Animal-level data		Model-predicted data	
	Continuous	Discrete	Continuous	Discrete
1. Incubation period				
Pan-serotype	Weibull(2.3,3.2)	**Binomial(8,0.4)	Logistic(4.1,0.4)	Binomial(6,0.7)
O	Lognormal(3.3,1.2)	Binomial(9,0.3)	Logistic(3.5,0.4)	**Binomial(5,0.73)
Asia1	Normal(3.2,1.1)	**Binomial(5,0.6)	Logistic(3.4,0.5)	**Binomial(5,0.7)
A	Normal(3.8,1.4)	**Binomial(7,0.5)	Logistic(4.4,0.2)	Binomial(6,0.77)
2. Latent duration				
Pan-serotype	Pert(1,1,3.5)	Binomial(3,0.4)	Pert(1.2,1.2,2.4)	Binomial(2,0.7)
O	Logistic(1.2,0.3)	Binomial(3,0.5)	Pert(1.4,1.5,2.4)	Binomial(2, 0.96)
Asia1	Normal(1.3,0.5)	**Binomial(2, 0.7)	Normal(1.4, 0.2)	Binomial(2, 0.8)
A	Pert(1,1,3.3)	Binomial(3,0.4)	Pert(1.2,1.2,1.7)	Binomial(2, 0.5)
3. Subclinical infectious duration				
Pan-serotype	Weibull(2,3.2)	**Poisson(2.4)	Weibull(6.8,4.6)	Binomial(5,0.6)
O	Logistic(1.6,0.7)	**Poisson(1.7)	Logistic(2.4,0.25)	Binomial(3,0.7)
Asia1	**Normal(1.8,0.9)	Binomial(3,0.6)	Logistic(2,0.3)	Binomial(3,0.66)
A	Logistic(2.7,0.8)	**Poisson(2.8)	Logistic(3.4,0.16)	Binomial(5,0.7)
4. Clinical infectious duration				
Pan-serotype	Pert(2.4,6.2,15.1)	**Poisson(7.07)	Pert(3.7,9.7,10)	Binomial(10,0.86)
O	**Normal(6.6,2.5)	**Poisson(6.6)	Pert(2.7,8.9,9)	Binomial(9,0.9)
*Asia1	Constant(5.8)	Constant(5.8)	Pert(3.2,9.9,10)	Binomial(10,0.86)
A	Pert(3.6,6.3,16.4)	**Poisson(7.5)	Normal(9.1,0.6)	Binomial(10,0.9)
5. Total infectious duration				
Pan-serotype	Pert(3.1,8.2,19)	**Poisson(9.2)	Normal(11.4,1.1)	Binomial(13,0.9)
O	Pert(4,4,26.3)	**Poisson(7.9)	Pert(6.8,10.7,10.7)	**Binomial(11,0.9)
*Asia1	Constant(7.8)	Constant(7.8)	Pert(6.4,11.9,12)	**Binomial(12,0.9)
A	Pert(7,7,25.6)	Poisson(10.16)	Pert(9.2,12.5,12.5)	Binomial(13,0.96)

* The data were not sufficient in number to derive a distribution. Constant distribution represents the mean durations for the respective virus serotypes.

**Anderson-Darling or Chi Square test showed a good fit (p>0.05).

Supplementary Table S5. The distribution fit for the FMD phase durations obtained from the animal-level data and best-fit AFT model data for non-threshold approach to define infectiousness. The animal-level data are from the non-censored animals only.

Serotypes	Animal-level data		Model-predicted data	
	Continuous	Discrete	Continuous	Discrete
1. Incubation duration				
The distribution fit for threshold was the same as the non-threshold defined infectiousness (Table 4).				
2. Latent duration				
Pan-serotype	Pert(1,1,3.2)	Binomial(3,0.4)	Logistic(1.3,0.07)	Binomial(2,0.5)
O	Logistic(1.05,0.14)	Binomial(2,0.57)	Logistic(1.3,0.09)	Binomial(2,0.6)
Asia1	Normal(1.3,0.5)	Poisson(1.3)	Logistic(1.2,0.1)	Binomial(2,0.6)
A	Pert(1,1,3.3)	Binomial(3,0.4)	Logistic(1.3,0.04)	Binomial (2,0.5)
3. Subclinical infectious duration				
Pan-serotype	Weibull(2.2,3.2)	Binomial(12,0.2)	Logistic(3.1,0.4)	Binomial(5,0.5)
O	Logistic(1.8,0.6)	Poisson(1.9)	Logistic(2.4,0.2)	Binomial(3,0.7)
Asia1	Normal(2,0.85)	**Binomial(3,0.66)	Normal(2.6,0.9)	Binomial(3,0.8)
A	Normal(2.8,1.5)	**Poisson(2.8)	Normal(3.4, 0.4)	Binomial(5,0.6)
4. Clinical infectious duration				
Pan-serotype	**Normal(8.8,3.4)	**Poisson(8.8)	Logistic(11.3,0.7)	Binomial(15,0.74)
O	**Normal(8.5,3.3)	**Poisson(8.5)	Logistic(10.4,0.9)	Binomial(15,0.68)
*Asia1	*Constant(6)	*Constant(6)	Logistic(10.7,1.1)	Binomial(17,0.63)
A	**Normal(9.4,3.5)	Poisson(9.4)	Logistic(11.6,0.3)	Binomial(15,0.8)
5. Total infectious duration				
Pan-serotype	Pert(4.6,8.8,26.3)	**Poisson(11.1)	Logistic(13.9,0.7)	Binomial(15,0.9)
O	**Normal(10.2,3.4)	**Poisson(10.2)	Logistic(12.9,0.6)	Binomial(14,0.9)
*Asia1	*Constant(8.3)	*Constant(8.3)	Pert(6.8,13.8,13.8)	**Binomial(14, 0.9)
A	Pert(5.5,9.6,26.8)	**Poisson(11.96)	Logistic(14.7,0.4)	Binomial(15,0.97)

* The data were not sufficient in number to derive a distribution. Constant distribution represent the mean durations for the respective virus serotypes.

**Anderson-Darling or Chi Square test showed a good fit (p>0.05).

References

Pacheco, J., Stenfeldt, C., Rodriguez, L., Arzt, J., 2016a. Infection dynamics of foot-and-mouth disease virus in cattle following intranasopharyngeal inoculation or contact exposure. Journal of comparative pathology 155, 314-325.

Pacheco, J.M., Gladue, D.P., Holinka, L.G., Arzt, J., Bishop, E., Smoliga, G., Pauszek, S.J., Bracht, A.J., O'Donnell, V., Fernandez-Sainz, I., Fletcher, P., Piccone, M.E., Rodriguez, L.L., Borca, M.V., 2013. A partial deletion in non-structural protein 3A can attenuate foot-and-mouth disease virus in cattle. Virology 446, 260-267.

Pacheco, J.M., Lee, K.N., Eschbaumer, M., Bishop, E.A., Hartwig, E.J., Pauszek, S.J., Smoliga, G.R., Kim, S.M., Park, J.H., Ko, Y.J., Lee, H.S., Tark, D., Cho, I.S., Kim, B., Rodriguez, L.L., Arzt, J., 2016b. Evaluation of Infectivity, Virulence and Transmission of FDMV Field Strains of Serotypes O and A Isolated In 2010 from Outbreaks in the Republic of Korea. PLoS One 11, e0146445.

Stenfeldt, C., Arzt, J., Pacheco, J.M., Gladue, D.P., Smoliga, G.R., Silva, E.B., Rodriguez, L.L., Borca, M.V., 2018a. A partial deletion within foot-and-mouth disease virus non-structural protein 3A causes clinical attenuation in cattle but does not prevent subclinical infection. Virology 516, 115-126.

Stenfeldt, C., Eschbaumer, M., Pacheco, J.M., Rekant, S.I., Rodriguez, L.L., Arzt, J., 2015. Pathogenesis of primary foot-and-mouth disease virus infection in the nasopharynx of vaccinated and non-vaccinated cattle. PLoS One 10, e0143666.

Stenfeldt, C., Eschbaumer, M., Rekant, S.I., Pacheco, J.M., Smoliga, G.R., Hartwig, E.J., Rodriguez, L.L., Arzt, J., 2016. The foot-and-mouth disease carrier state divergence in cattle. J Virol 90, 6344-6364.

Stenfeldt, C., Hartwig, E., Smoliga, G., Palinski, R., Silva, E.B., Bertram, M., Fish, I.H., Pauszek, S.J., Arzt, J., 2018b. Contact Challenge of Cattle with Foot-and-Mouth Disease Virus Validates the Role of the Nasopharyngeal Epithelium as the Site of Primary and Persistent Infection. MSphere.
